# The Retrospective Review of the Conversion of Ecmo Mode in Pediatric Patients: A Single Center Experience in Japan

**DOI:** 10.1186/2197-425X-3-S1-A494

**Published:** 2015-10-01

**Authors:** T Niitsu, T Sueishi, I Watanabe, M Motomura, Y Nakayama, O Saito, N Shimizu

**Affiliations:** Tokyo Metropolitan Children's Medical Center, Department of Paediatric Emergency and Critical Care Medicine, Tokyo, Japan

## Introduction

Recently the indication of extracorporeal membrane oxygenation (ECMO) treatment has been expanded to various diseases with circulatory and respiratory failure and the duration of ECMO management tend to be longer due to the development of related device. in some cases with long run of ECMO, the conversion of ECMO needs to be considered depending on the respiratory and circulatory status.

## Purpose

To evaluate the characteristics of the episodes of ECMO mode conversion.

## Methods

Retrospective observational study of ECMO patients admitted to the PICU at Tokyo Metropolitan Children's Medical Center between March 2010 and December 2014.

## Results

Of fifty eight patients with ECMO treatment during this period, seven patients needed to convert the ECMO mode. Among these seven patients, the indication of ECMO management was postsurgical respiratory failure in five patients and respiratory failure due to infection (RS virus, pertussis) in two patients. Three patients initially on VV-ECMO need to be converted to VA-ECMO due to hypotension and two patients on VA-ECMO were converted to VV-ECMO due to persistent respiratory failure. in one patient, ECMO mode was changed from VV-ECMO to VA-ECMO because of pulmonary hypertension, but returned to VV-ECMO with the control of PH. Another patient had the conversion from VA to VV-ECMO and needed to return to VA-ECMO due to septic shock. the procedure of cannulation was performed by the surgical team and complications related to the cannula change were seen in one patient with hypotension and ventricular arrhythmia. the duration of ECMO management was longer in the patients with the change of ECMO mode compared to those without the conversion of ECMO mode (13 days vs. 29 days, p < 0.01). All of these seven patients could wean off ECMO treatment and three of them survived to discharge (survival rate of 43%).

## Conclusions

The conversion of ECMO mode in response to the change of respiratory and circulatory status might be an appropriate option in the management of pediatrics ECMO patients.

